# Population structure of *Aphyocypris normalis*: phylogeography and systematics

**DOI:** 10.3897/zookeys.872.33105

**Published:** 2019-08-26

**Authors:** Xiao-Xia Huang, Kui-Ching Hsu, Bin Kang, Po-Hsun Kuo, Wan-Hsin Tsai, Chih-Ming Liang, Hung-Du Lin, Wei-Kuang Wang

**Affiliations:** 1 Key laboratory of atmospheric environment and processes in the boundary layer over the low-latitude plateau region, Yunnan University, Kunming, 650091, China Yunnan University Kunming China; 2 College of Fisheries, Guangdong Ocean University, Zhanjiang 524088, China Guangdong Ocean University Zhanjiang China; 3 College of Fisheries, Ocean University of China, Qingdao 266003, China Ocean University of China Qingdao China; 4 Department of Industrial Management, National Taiwan University of Science and Technology, Taipei 106, Taiwan National Taiwan University of Science and Technology Taipei Taiwan; 5 Department of Environmental Engineering and Science, Feng Chia University, Taichung 407, Taiwan Feng Chia University Taichung Taiwan; 6 The Affiliated School of National Tainan First Senior High School, Tainan 701, Taiwan The Affiliated School of National Tainan First Senior High School Tainan Taiwan

**Keywords:** Dispersal, Hainan Peninsula, Vicariance, Wuzhishan and Yinggeling Mountains Range

## Abstract

*Aphyocypris normalis* (Teleostei: Cyprinidae) is an endemic species in South China, but little is known about its genetic structure. This study examined the population structure of *A. normalis* using sequences of the mitochondrial DNA control region and cytochrome *b* gene (2,086 bp). In total, 107 specimens were collected from nine populations. All 105 mtDNA haplotypes were identified as belonging to two allopatric phylogroups. The results of a statistical dispersal-vicariance analysis (S-DIVA) suggested that the ancestral populations of *A. normalis* were distributed widely on Hainan Island and east of the Leizhou Peninsula. A comparison of the fixation indices N_ST_ (0.532) and G_ST_ (0.004) revealed that the phylogeny and geography had a significant relationship. Our study found that (1) the Wuzhishan and Yinggeling Mountain Range was an important barrier limiting gene exchange between populations on both sides; (2) cyclic climate changes may have shaped migrations and population differentiations; and (3) different colonization times caused different population diversities between codistributed species. In addition, the inter- and intraspecific diversities of the genus *Aphyocypris* were estimated.

## Introduction

Hainan Island is located in the transitional zone between tropical and temperate zones. The island is separated from mainland China to the north by the Qiongzhou Strait and from mainland Vietnam to the west by the Gulf of Tonkin. Hainan Island was first isolated from the mainland by the results of volcanism and rising sea levels approximately 2–2.5 million years ago (mya) (e.g., Zeng and Zeng 1989; Zhao et al. 2007). Based on the ichthyofaunal similarities (Li 1981), Hainan Island was defined as a unit subregion in the South China region. Our study found that some freshwater fishes, e.g., *Glyptothorax hainanensis* and *Opsariichthys hainanensis*, are considered endemic to Hainan Island, but previous studies (Chen et al. 2007; Lin et al. 2016) have found that these two species are also distributed in the Pearl River subregion. Moreover, geological evidence indicates that land bridges repeatedly connected the island to the Asian continent during the Pleistocene glaciations (Voris 2000; Zhao et al. 2007). According to phylogeographic studies of freshwater fishes (e.g., genus *Glyptothorax* see Chen et al. 2007; *Garra orientalis* see Yang et al. 2016; genus *Opsariichthys* see Lin et al. 2016), during Pleistocene glaciations, migrants probably moved between mainland China and island populations. The gene flows were interrupted when Hainan Island was separated from mainland China by the sinking of the Qiongzhou Strait (Morton and Blackmore 2001; Yap 2002).

**Figure 1.**
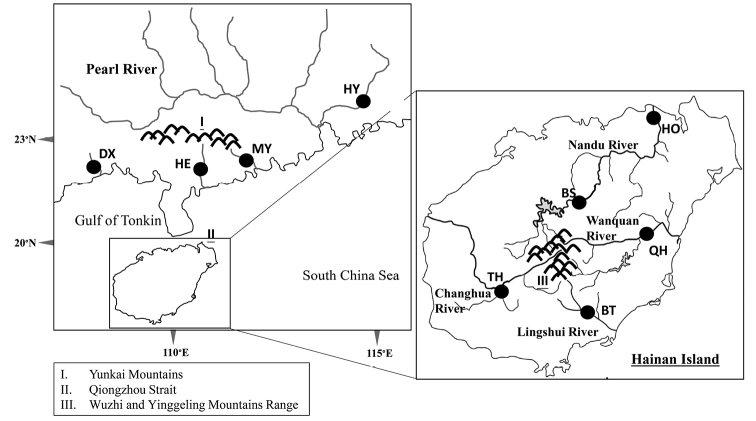
*Aphyocypris normalis* sampling locations on Hainan Island and adjacent areas. All localities sampled in this study are indicated by •.

The Wuzhishan and Yinggeling Mountain Range (WY Range) rises steeply from the central and southern regions of Hainan Island and gives way to a broad plain in the north. Lin et al. (2010) found that the WY Range was an important barrier based on the population structure of the Reeves’s butterfly lizard. However, Huang et al. (2013) suggested that WY Range did not act as a barrier to gene flow among populations of oriental garden lizards (*Calotes versicolor*). In addition, the four largest rivers on the island, the Nandu, Changhua, Wanquan, and Linshui Rivers, originate from the WY Range and flow outwards into the Qiongzhou Strait, South China Sea, and Gulf of Tonkin, respectively (Fig. 1). According to the landforms, the WY Range isolated these four rivers into two regions, southern Hainan (Wanquan and Linshui rivers) and northern Hainan (Nandu and Changhua Rivers). Yang et al. (2016) and Zhou et al. (2017) proposed that the WY Range was an important phylogeographic break based on the population structures of the cyprinid fishes *G. orientalis* and *Onychostoma lepturum*. However, Lin et al. (2016) suggested that the WY Range did not act as a barrier to gene flow among populations of another cyprinid, *O. hainanensis*.

*Aphyocypris normalis* is a small cyprinid fish restricted to Hainan Island and its adjacent area. Thus, this species is an ideal freshwater fish species to study the biological consequences of the geological history of river systems in this area. *Aphyocypris normalis* was named *Nicholsicypris normalis* until 2011. Liao et al. (2011) suggested that *Pararasbora*, *Nicholsicypris*, and *Yaoshanicus* were synonyms of *Aphyocypris* based on previous morphological studies (Chu 1935; Tzeng 1986; Kottelat 2001). Although Tang et al. (2010) supported their close interrelationship with molecular data, the genetic distances among them were not estimated. Thus, our study examined the taxonomic status of *Pararasbora*, *Nicholsicypris*, *Yaoshanicus*, and *Aphyocypris* based on molecular data. In addition, the species diversity within *Aphyocypris* is very low. Liao et al. (2011) found that there are two species within *Aphyocypris* (*A. chinensis* and *A. kikuchii*), and *Pararasbra* (*P. moltrechti*), *Nicholsicypris* (*N. normalis*), and *Yaoshanicus* (*Y. arcus*) are monotypic. Until now, there were eight species within the genus *Aphyocypris* (Froese and Pauly 2018), and new species were described (Zhu et al. 2013). Thus, our study examined the diversity within *Aphyocypris*.

The major questions in our study are as follows: (1) What are the taxonomic boundaries within *Aphyocypris* and its close relatives? (2) How did *A. normalis* colonize the rivers on the island and mainland? (3) Is there a phylogeographic break in freshwater fish on Hainan Island? To address the aforementioned questions, the mitochondrial DNA cytochrome *b* gene and control region sequences (hereafter mtDNA) were used to evaluate the phylogenetic relationships and population genetic structure (e.g., Chen et al. 2007; Yang et al. 2012, 2016; Chiang et al. 2010, 2013; Hsu et al. 2014). These results will help to establish mtDNA sequence data and develop conservation strategies for freshwater fishes on Hainan Island.

## Materials and methods

### Sampling and molecular methods

A total of 107 specimens of *A. normalis* was collected from nine localities on Hainan Island and mainland China (Fig. 1, detailed in Table 1). These populations were sorted into five groups based on the landforms. Fishes were collected from field sites with seines and euthanized with MS-222 (Sigma). Samples were fixed and stored in 100% ethanol. Genomic DNA was extracted from muscle tissue using a Genomic DNA Purification Kit (Gentra Systems, Valencia, CA). The entire cyt b gene and control region fragment were amplified by polymerase chain reaction (PCR) using primers from Xiao et al. (2001) and Zhou et al. (2017), respectively. Each 50 µl PCR mixture contained 5 ng template DNA, 5 µl 10x reaction buffer, 5 µl dNTP mix (10 mM), 5 pmol of each primer and 2 U of Taq polymerase (Promega, Madison, WI, U.S.A.). PCR was performed on an MJ Thermal Cycler with the following program: one cycle of denaturation at 95 °C for 4 min, 30 cycles of denaturation at 94 °C for 45 s, annealing at 48 °C for 1 min 15 s and extension at 72 °C for 1 min 30 s, followed by 72 °C extension for 10 min and sample storage at 4 °C. The purified PCR products were sequenced using an ABI 377 automated sequencer (Applied Biosystems, Foster City, CA, U.S.A.). Chromatograms were checked with the Chromas software (Technelysium), and sequences were manually edited using BioEdit 6.0.7 (Hall 1999). Moreover, to examine the taxonomic status among *Pararasbora*, *Yaoshanicus*, *Nicholsicypris* and *Aphyocypris*, the sequences of Opsariichthyinae from GenBank were obtained (see Results).

**Table 1.** Sampling locations, abbreviation (Abbr.) and summary statistics of *Aphyocypris normalis*. Haplogroups correspond to the haplogroups recover in BEAST phylogeny (Fig. 3).

**River**	**Locations (Abbr.)**	**Latitude / Longitude**	**Sample size**	**Haplotype diversity (h)**	**Nucleotide diversity (%)**		**phylogroup**
					**θ_π_**	**θ_ω_**	
**North of Yunkai Mountains**							
Dongjiang	Heyuan (HY)	23°44'N, 114°42'E	14	0.99	0.23	0.32	I
**South of Yunkai Mountains**							
**East of Leizhou Peninsular**							
Moyangjiang	Yangchung (MY)	22°26'N, 111°56'E	11	1.00	0.61	0.71	I
Jianjiang	Hexi (HE)	22°21'N, 110°56'E	14	1.00	1.12	1.16	I
**West of Leizhou Peninsular**							
Beilun River	Dongxing (DX)	21°32'N, 107°58'E	7	1.00	1.08	1.26	I
**Hainan Island**							
**Northern**							
Nandu River	Haikou (HO)	19°57'N, 110°19'E	13	1.00	0.97	1.23	I
Wanquan River	Qionghai (QH)	19°09'N, 110°18'E	8	1.00	1.43	1.68	I
Zhubijang	Basha (BS)	19°13'N, 109°26'E	11	1.00	0.85	0.97	I
**Southern**							
Lingshui River	Baoting (BT)	18°38'N, 109°42'E	20	0.99	0.50	0.52	II
Changhua River	Thanhuna (TH)	18°46'N, 109°30'E	9	1.00	0.68	0.78	II
**Total**				**107**	**1.00**	**1.49**	**2.45**

### Sequence alignment and phylogenetic inference

Nucleotide sequences were aligned in Clustal X 1.81 (Thompson et al. 1997). Levels of intrapopulation genetic diversity were estimated by indices of haplotype diversity (*h*) (Nei and Tajima 1983) and nucleotide diversity (θ_π_ and θ_ω_) (Jukes and Cantor 1969) in DnaSP 4.10.8 (Rozas et al. 2003). Comparing estimates generated by these two indices (θ_π_, current genetic diversity, and θ_ω_, historical diversity) provides insight into population dynamics over recent evolutionary history (Templeton 1993). The existence of phylogeographic structure was examined following Pons and Petit (1996) by calculating two genetic differentiation indices, G_ST_ and N_ST_, in DnaSP. Pairwise F_ST_ values implemented by DnaSP were used to examine the spatial partitioning of genetic variation among populations. AMOVA (analysis of molecular variance) partitioned were used to the observed variation among samples into within-population (F_ST_), within-group (F_SC_) and among-group (F_CT_) components in Arlequin version 3.5 (Excoffier and Lischer 2010). Arlequin was also used to construct the haplotype network. The program SAMOVA (Dupanloup et al. 2002) was used to identify groups of adjacent sampling locations with the maximum extent of genetic differentiation. These analyses used 500 simulated annealing steps and compared maximum indicators of differentiation (F_CT_) when the program was instructed to identify K = 2 to K = 8 partitions of the sampling area.

A phylogenetic tree was created by BEAST 1.8.0 (Drummond et al. 2013), which is a Bayesian statistical framework. Phylogenetic relationships were also inferred using maximum-likelihood (ML) in MEGA 6 (Tamura et al. 2013). The GTR+I+G substitution model was selected using the Akaike information criteria (AICc) in jModelTest 2.0 (Darriba et al. 2012). The time to the most recent common ancestor (T_MRCA_) was also calculated with the software package BEAST. A molecular clock was calibrated using a divergence rate of 2% per million years (Yang et al. 2016). We used a strict clock with a Bayesian skyline tree and estimated the divergence times of the major lineages to the most recent common ancestor (T_MRCA_). We ran 10^6^ generations. The output was visualized in Tracer v1.6 (Rambaut et al. 2013) to verify that convergence and suitable effective sample size were achieved for all parameters. Burn-in and plots for each analysis were visualized using Tracer. The TREEANNOTATOR in the BEAST package was used to summarize tree data, and the tree was viewed using FigTree v1.3 (Rambaut 2014). For ML analysis, bootstrapping was performed with 1000 replications.

In addition, to determine the possible diversification scenarios of *A. normalis*, a statistical dispersal-vicariance analysis (S-DIVA), a program that complements DIVA, was employed to determine statistical support for ancestral range reconstructions (Yu et al. 2010). The tree file formats were generated by the program BEAST with 10^7^ Markov Chain Monte Carlo (MCMC) steps and the first 10% as burn-in. The analysis was performed using the ‘maxareas = 5’ option because the populations were divided into six groups (Table 1).

## Results

### Taxonomic status

**Figure 2.**
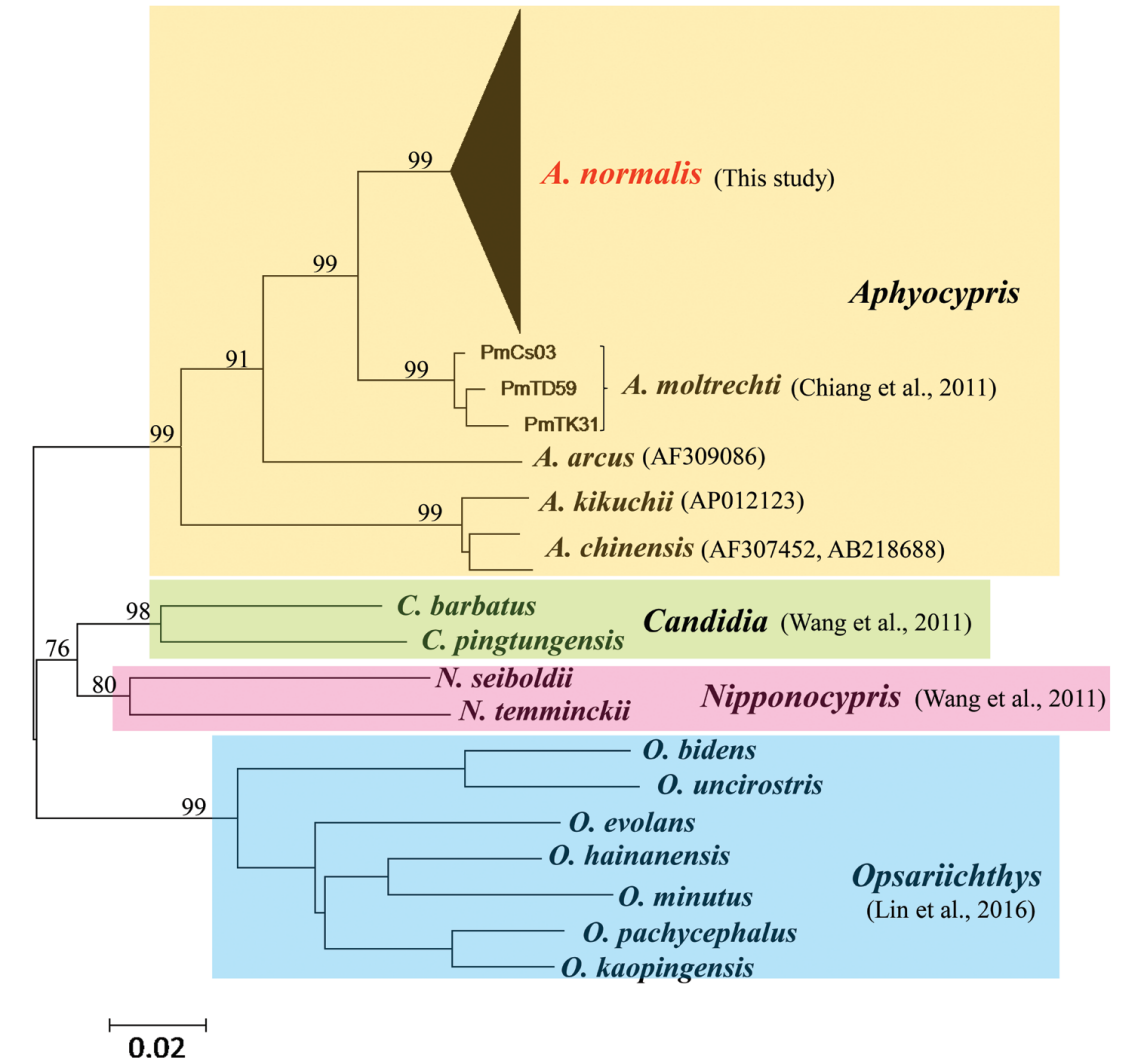
Phylogenetic relationships of the genera *Aphyocypris*, *Candidia*, *Nipponocypris*, and *Opsariichthys* using ML analyses of cyt b gene sequence data. Numbers along the branches indicate the percentage of bootstrap support obtained in the ML analyses.

Although the genus *Aphyocypris* was included in Danioninae, Liao et al. (2011) reassigned this genus into Opsariichthyinae. Thus, sequences of Opsariichthyinae in GenBank from Chiang et al. (2011), Wang et al. (2011) and Lin et al. (2016) were downloaded (Fig. 2). The ML tree (Fig. 2) showed that all sequences fell into four monophyletic groups corresponding to four genera, *Aphyocypris*, *Candidia*, *Nipponocypris*, and *Opsariichthys*. Among these four genera, the mean pairwise p-distance was 15.84%, with a range of 12.39% to 18.22%. The range of the divergences within these genera was 3.15% to 11.40% (Table 2). In addition, three *Aphyocypris* species, *A. normalis*, *A. moltrechti*, and *A. chinensis*, are also monophyletic groups. The mean intraspecific divergence within these *Aphyocypris* species was 1.91%. The mean interspecific p-distance was 9.89%, with a range of 2.63% to 12.59% (Table 2).

**Table 2.** Pairwise p-distance (%) within and among genera and species.

	**within**	**A2**	**A3**	**A4**	**A5**	**B**	**B2**	**C**	**C2**	**D**	**D2**	**D3**	**D4**	**D5**	**D6**	**D7**
***A. Aphyocypris***	**3.15**					**15.31**		**16.02**		**18.22**						
A1. *A. normalis*	2.28	5.78	9.74	12.59	12.52											
A2. *A. moltrechti*	1.11		8.92	11.78	11.64											
A3. *A. arcus*	–			11.67	11.58											
A4. *A. kikuchii*	–				2.63											
A5. *A. chinensis*	2.33															
***B. Candidia***	**8.77**							**12.39**		**16.91**						
B1. *C. barbatus*	–						8.77									
B2. *C. pingtungensis*	–															
***C. Nipponocypris***	**11.40**									**16.17**						
C1. *N. seiboldii*	–								11.4							
C2. *N. temminckii*	–															
***D. Opsariichthys***	**10.69**															
D1. *O. bidens*											6.58	12.81	13.25	14.21	12.54	12.46
D2. *O. uncirostris*												13.42	13.33	13.95	13.42	12.63
D3. *O. evolans*													9.21	9.47	9.74	9.12
D4. *O. hainanensis*														7.28	9.47	9.39
D5. *O. minutus*															8.60	9.39
D6. *O. pachycephalus*																4.30
D7. *O. kaopingensis*																

### Genetic diversity of *A. normalis*

The complete 1,141 base pairs (bp) of cyt b (MH909846-MH909955) and 945 bp of the control region (MH909956-MH910020) sequences were analyzed. A total of 105 haplotypes (2,086 bp, 270 variable sites and 170 phylogenetic informative sites) were obtained for 107 sequences from nine populations analyzed (Table 1; Fig. 1). Nucleotide sequences were A+T rich (64.9%). Haplotype diversities within populations ranged from 0.99 to 1.00 (Table 1). The nucleotide diversity (θ_π_) in the region was the highest in northern Hainan Island (average = 1.20) and the lowest in the northern Yunkai Mountains (population HY, 0.23). At the population level, the nucleotide diversity (θ_π_) was the highest in population QH (northern Hainan Island; 1.43) and the lowest in population HY (north of Yunkai Mountains; 0.23). Estimates of the current (θ_π_) and historical (θ_ω_) genetic diversity per site for each sample indicated that all samples showed a pattern of decline (θ_π_ < θ_ω_; Table 1).

### Population structure and history

All mtDNA haplotypes were population-private haplotypes. Our study found that the sequences were unique, excluding two identical sequences within populations BT and HY. Geographical division assessed by DnaSP indicated significant differentiation among populations (F_ST_ = 0.530). The pairwise F_ST_ values ranged from 0.144 (between populations HO and QH) to 0.820 (between populations HY and BT) (Table 3). After pooling by region (Table 1), the pairwise F_ST_ within the mainland region (mean F_ST_ = 0.493) was smaller than that within island regions (mean F_ST_ = 0.514) (Table 3). The mean F_ST_ between the mainland and island was similar to that within the island (Table 3). A comparison of the fixation indices N_ST_ (0.532) and G_ST_ (0.004) revealed that N_ST_ was much larger than G_ST_. This result suggested a strong relationship between phylogeny and geography. These results indicated that the population differentiations were significant.

**Table 3.** Matrix of pair-wise *F*_ST_ (below diagonal) and p values (above diagonal) based on mtDNA in *N. normalis*. Refer to Table 1 for the abbreviations of localities.

	**MY**	**HE**	**DX**	**HO**	**QH**	**BS**	**BT**	**TH**
**HY**	0.67	0.57	0.62	0.56	0.49	0.72	0.82	0.79
**MY**	0.00	0.38	0.45	0.37	0.29	0.59	0.71	0.67
**HE**	0.00	0.00	0.27	0.26	0.18	0.46	0.61	0.57
**DX**	0.00	0.00	0.00	0.29	0.18	0.52	0.64	0.57
**HO**	0.00	0.00	0.00	0.00	0.14	0.51	0.61	0.58
**QH**	0.00	0.00	0.00	0.00	0.00	0.41	0.54	0.50
**BS**	0.00	0.00	0.00	0.00	0.00	0.00	0.73	0.69
**BT**	0.00	0.00	0.00	0.00	0.00	0.00	0.00	0.44

**Figure 3.**
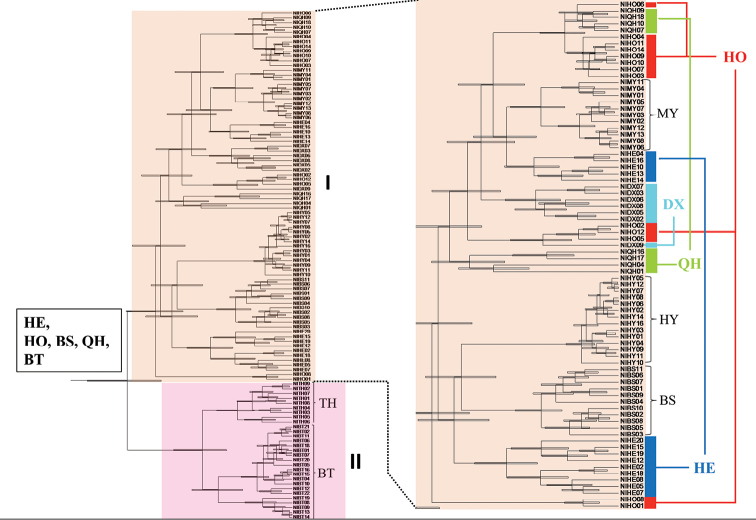
BEAST-derived chronograms of 107 mitochondrial DNA sequences of *Aphyocypris normalis*. The S-DIVA analysis graphical representation of the ancestral distribution is given in the box above the node.

The Bayesian phylogenetic tree is shown in Figure 3 and is divided into two major phylogroups (I and II). Phylogroup I included seven populations in northern Hainan Island (including) and phylogroup II included only two populations in southern Hainan Island. The best SAMOVA grouping schemes partitioned the sampling area into five groups (K = 5; F_CT_ = 0.35, p < 0.001). All samples were divided into five units: (1) BS, (2) BT, (3) TH, (4) HY and (5) others. The results of AMOVA indicated significant genetic structures at several levels (Table 4), but the most genetic variability was accounted for by between-group differences among five SAMOVA units. Among these five groups, 34.53%, 24.26% and 41.21% variations existed among groups, among populations within groups and within populations, respectively.

**Table 4.** Analysis of molecular variance (AMOVA).

**Scheme**	**Category description**	% **Var.**	**Statistic**	**P**
1. Two geological groups (HY+MY+HE +DX) (HO+QH+BS+TH+BT)				
	Among regions	4.54	*F*_SC_ = 0.54	<0.001
	Among populations in region	51.64	*F*_ST_ = 0.56	<0.001
	Within population	43.82	*F*_CT_ = 0.05	<0.001
2. Three geological groups (HY) (MY+HE +DX) (HO+QH+BS+TH+BT)				
	Among regions	4.65	*F*_SC_ = 0.54	<0.001
	Among populations in region	51.37	*F*_ST_ = 0.56	<0.001
	Within population	43.98	*F*_CT_ = 0.05	<0.001
3. Four geological groups (HY) (MY+HE) (DX) (HO+QH+BS+TH+BT)				
	Among regions	-0.16	*F*_SC_ = 0.55	<0.001
	Among populations in region	55.41	*F*_ST_ = 0.55	<0.001
	Within population	44.75	*F*_CT_ = -0.00	<0.01
4. Four geological groups (HY) (MY+HE +DX) (HO+QH+BS) (TH+BT)				
	Among regions	18.82	*F*_SC_ = 0.39	<0.001
	Among populations in region	32.03	*F*_ST_ = 0.51	<0.001
	Within population	49.14	*F*_CT_ = 0.19	<0.01
5. Two phylogroups (HY+HE+MY+DX+QH+BS+HO) (TH+BT)				
	Among regions	31.03	*F*_SC_ = 0.46	<0.001
	Among populations in region	31.94	*F*_ST_ = 0.63	<0.001
	Within population	37.03	*F*_CT_ = 0.31	<0.01
6. Five SAMOVA groups (HY) (HE+MY+DX+QH+HO) (BS) (TH) (BT)				
	Among regions	**34.53**	*F*_SC_ = 0.37	<0.001
	Among populations in region	24.26	*F*_ST_ = 0.59	<0.001
	Within population	41.21	*F*_CT_ = **0.36**	<0.001

The coalescence time of *A. normalis* was estimated to be in the early Pleistocene (T_MRCA_= 2.33 mya, 1.92 – 2.74). The results of the S-DIVA analysis indicated that possible ancestral populations of *A. normalis* were distributed on Hainan Island and east of the Leizhou Peninsula. The rising of the WY Range on Hainan Island separated the populations into two groups, south and north of the WY Range. After a vicariance event, the populations in the north of the WY Range migrated to the west of the Leizhou Peninsula.

## Discussion

### Inter- and interspecific diversities within *Aphyocypris*

Today, the genera *Pararasbora*, *Yaoshanicus*, and *Nicholsicypris* are synonymous with the genus *Aphyocypris*. Our study considered that a genus should fulfill two criteria at least, monophyly and distinctness. In this study, the range of the p-distance among the genera *Candidia*, *Nipponocypris* and *Opsariichthys* was 12.39% to 16.17% (mean = 15.16%) (Table 2). The range of the p-distance between the genus *Aphyocypris* and the other three genera ranged from 15.31% to 18.22% (mean = 16.52%) (Table 2). Moreover, the mean interspecific p-distance within the genus *Aphyocypris* was 9.89%, with a range of 2.63% to 12.59% (Table 2). The mean interspecific p-distance within the genus *Opsariichthys* was 10.69%, with a range of 4.30% to 14.21%. Thus, our study determined that the genera boundary was 15.00%. Based on the genetic variations, our study supported the synonymization of *Pararasbora* (*P. moltrechti*), *Yaoshanicus* (*Y. arcus*) and *Nicholsicypris* (*N. normalis*) with *Aphyocypris* (Liao et al. 2011). Moreover, Tzeng (1986) indicated that *A. normalis* is conspecific with *A. moltrechti*, but our results did not support that finding (Fig. 2; Table 2).

### Phylogeography of *Aphyocypris normalis*

The geological studies proposed that approximately 2–2.5 mya, Hainan was first isolated from the mainland by the results of volcanism and the ground fall in the current Qiongzhou Strait (e.g., Zeng and Zeng 1989). During the Pleistocene glaciations, migrants probably moved between mainland China and the island by the sinking of the Gulf of Tonkin and Qiongzhou Strait (Morton and Blackmore 2001; Yap 2002). Previous studies (Chen et al. 2007; Lin et al. 2016; Yang et al. 2016) have indicated that during the Pleistocene glaciations, the sea level dropped, and the entire region of the Gulf of Tonkin and the Qiongzhou Strait became part of the coastal plain of the Asian continent (Fig. 1). The strait and exposed continental shelves assisted in population dispersions. Thus, the gene flow among the ancestral populations of Hainan Island and the adjacent areas were unlimited. The results presented here suggest that the ancestral populations of *A. normalis* were distributed widely south of the Yunkai Mountains, including Hainan Island, and east of the Leizhou Peninsula in the early Pleistocene.

Subsequently, our results found that the populations on southern Hainan Island (TH and BT) diverged by a vicariance event. At 0.458 mya, the WY Range arose on Hainan Island and isolated the population in the southern Hainan Island region as lineage II (Fig. 3). This result agrees with previous studies (Yang et al. 2016; Zhou et al. 2017). The WY Range is located in the central and southern regions of Hainan Island and approaches an elevation of 1800 m. The landforms reflect that the rivers on island originate from the central mountainous area and flow outwards. Due to the steep topology of the island, most rivers run directly into the oceans with no connection to the neighboring drainage systems. As in previous phylogeographic studies of freshwater fishes (Yang et al. 2016; Zhou et al. 2017), the WY Range was an important barrier. Zhou et al. (2017) found that the populations of the cyprinid fish *O. lepturum* on Hainan Island could be divided into three units based on their location in the Qiongzhou Strait, the Gulf of Tonkin and the South China Sea. The genetic structure of *A. normalis* on Hainan Island showed the same pattern as that of *O. lepturum* (Zhou et al. 2017). Thus, the WY Range is a phylogeographic break in the phylogeography of *A. normalis*.

Based on previous studies (Chen et al. 2007; Lin et al. 2016; Yang et al. 2016), the populations on Hainan Island and the mainland diverged by a vicariance event, the Qiongzhou Strait. Previous studies (Chen et al. 2007; Lin et al. 2016; Yang et al. 2016) proposed that populations of freshwater fishes migrated from one side of the exposed Qiongzhou Strait to the other, and the populations of the island and the mainland separated into two highly divergent clades due to the rising sea level. Our study found that the populations of *A. normalis* on both sides of the Qiongzhou Strait were a mixture (Fig. 3). Although previous studies (Yap 2002; Zhou et al. 2017) considered that the Qiongzhou Strait is a phylogeographic break of freshwater fishes, we found that the populations of *G. orientalis* (Yang et al. 2016) and *O. hainanensis* (Lin et al. 2016) on both sides of the Qiongzhou Strait were mixed to some extent. In the population structure of *G. orientalis* (Yang et al. 2016), lineage I was only distributed to the north of Qiongzhou Strait, but lineage II was distributed both to the north and south of the strait. Likewise, the population structure of *O. hainanensis* (Lin et al. 2016) displayed the same pattern as that of *G. orientalis* (Yang et al. 2016). Moreover, geological studies (Voris 2000; Shi et al. 2006; Zhao et al. 2007) suggested that land bridges repeatedly connected the island to the Asian continent. Yang et al. (2016) proposed that when glaciation occurred, the lineages mixed. Thus, the phylogenetic analyses of *A. normalis* (Fig. 3) in the current study revealed a mixed genetic structure across the strait due to cyclic climate changes. When glaciation occurred again after the WY Range rose, some populations retreated from the mainland to the island. Thus, the phylogenetic analysis (Fig. 3) displayed two divergent phylogroups before the divergences among haplotypes included in the ancestral populations.

Our study found that *A. normalis* (this study) and *G. orientalis* (Yang et al. 2016) have similar distribution patterns and that these two species displayed similar phylogeographic patterns. Thus, our study suggested that the effects of environmental changes on Hainan Island and its adjacent area were general patterns. However, the populations of *G. orientalis* showed moderate to high genetic differentiation, but those within *A. normalis* showed a high level of genetic differentiation (Table 2). Although these two species both displayed differentiation, there was no shared haplotype between mainland and island populations, and there was no shared haplotype within *A. normalis*. The haplotype and nucleotide diversities of *A. normalis* are higher than those of *G. orientalis*. Moreover, the genus *Aphyocypris* only includes ten species in East Asia, but the genus *Garra* contains 138 species in Asia and Africa (Froese and Pauly 2018). Our study found that *A. normalis* and *G. orientalis* had different colonization times, with *A. normalis* in the early Pleistocene and *G. orientalis* in the late Pleistocene. Our study considered that the ancestral populations of these two species originated from different mainland populations. Thus, our future aim is to understand the comparative phylogeography and geological history in Hainan Island and its adjacent area.

## References

[B1] Chen XL Chiang TY Lin HD Zheng HS Shao KT Zhang Q Hsu KC (2007) Mitochondrial DNA phylogeographyof *Glyptothorax fokiensis* and *Glyptothorax hainanensis* in Asia. Journal of Fish Biology 70: 75–93. 10.1111/j.1095-8649.2007.01370.x

[B2] Chiang TY Lee TW Hsu KC Kuo CH Lin DY Lin HD (2011) Population structure in the endangered cyprinid fish *Pararasbora moltrechti* in Taiwan, based on mitochondrial and microsatellite DNAs. Zoological Science 28: 642–651. 10.2108/zsj.28.642 21882952

[B3] Chiang TY Lin HD Shao KT Hsu KC (2010) Multiple factors have shaped the phylogeography of Chinese spiny loach (*Cobitis sinensis)* in Taiwan as inferred from mitochondrial DNA variation. Journal of Fish Biology 76: 1173–89. 10.1111/j.1095-8649.2010.02589.x 20409169

[B4] Chiang TY Lin HD Zhao J Kuo PH Lee TW Hsu KC (2013) Diverse processes shape deep phylogeographical divergence in *Cobitis sinensis* (Teleostei: Cobitidae) in East Asia. Journal of Zoological Systematics and Evolutionary Research 51: 316–326. 10.1111/jzs.12030

[B5] Chu YT (1935) Comparative studies on the scales and on the pharyngeal and their teeth in Chinese cyprinids, with particular reference to taxonomy and evolution. Biol. Bull. St. John’s Univ. Shanghai 2, 1–225. 10.2307/1436747

[B6] Darriba D Taboada GL Doallo R Posada D (2012) jModelTest 2: more models, new heuristics and parallel computing. Nature Methods 9: 772. 10.1038/nmeth.2109 PMC4594756 22847109

[B7] Drummond AJ Rambau A Suchard M (2013) BEAST 1.8.0. http://beast.bio.ed.ac.uk

[B8] Dupanloup I Schneidera S Excoffier NL (2002) A simulated annealing approach to define the genetic structure of populations. Molecular Ecology 11: 2571–2581. 10.1111/j.1755-0998.2010.02847.x 12453240

[B9] Excoffier L Lischer HEL (2010) Arlequin suite version 3.5: a new series of programs to perform population genetics analyses under Linux and Windows. Molecular Ecology Resources 10: 564–567. 10.1111/j.1755-0998.2010.02847.x 21565059

[B10] Froese R Pauly D (2018) FishBase. World Wide Web electronic publication. http://www.fishbase.org

[B11] Hall TA (1999) BioEdit: a user-friendly biological sequence alignment editor and analysis program for Windows 95/98/NT. Nucleic Acids Symposium Series 41: 95–98.

[B12] Hsu KC Bor H Lin HD Kuo PH Tan MS Chiu YW (2014) Mitochondrial DNA phylogeography of *Semisulcospira libertina* (Gastropoda: Cerithioidea: Pleuroceridae): implications the history of landform changes in Taiwan. Molecular Biology Reports 41: 3733–3743. 10.1007/s11033-014-3238-y 24584517

[B13] Huang Y Guo X Ho SYW Shi H Li J Li J Cai B Wang Y (2013) Diversification and demography of the oriental garden lizard (*Calotes versicolor*) on Hainan Island and the adjacent mainland. PlosOne 8: e64754. 10.1371/journal.pone.0064754 PMC3694074 23840304

[B14] Jukes TH Cantor CR (1969) Evolution of protein molecules. In: Monro HN (ed.) , Mammalian protein metabolism.Academic Press, New York, NY, 21–132. 10.1016/B978-1-4832-3211-9.50009-7

[B15] Kottelat M (2001) Freshwater fishes of northern Vietnam. Washington DC: World Bank, 123 pp.

[B16] Li SZ (1981) Studies on zoogeographical divisions for fresh water fishes of China. Science Press, Beijing, China (Chinese).

[B17] Liao TY Kullander SO Lin HD (2011) Synonymization of *Pararasbora*, *Yaoshanicus*, and *Nicholsicypris* with *Aphyocypris*, and Description of a new species of *Aphyocypris* from Taiwan (Teleostei: Cyprinidae). Zoological Studies 50: 657–664.

[B18] Lin HD Kuo PH Wang WK Chiu YW Ju YM Lin FJ Hsu KC (2016) Speciation and differentiation of the genus *Opsariichthys* (Teleostei: Cyprinidae) in East Asia. Biochemical Systematics and Ecology 68: 92–100. 10.1016/j.bse.2016.07.001

[B19] Lin LH Ji X Diong CH Du Y Lin CX (2010) Phylogeography and population structure of the Reevese’s butterfly lizard (*Leiolepis reevesii*) inferred from mitochondrial DNA sequences. Molecular phylogenetics and Evolution 56: 601–607. 10.1016/j.ympev.2010.04.032 [Epub 2010 Apr 28.] 20433932

[B20] Morton B Blackmore G (2001) South China Sea. Marine Pollution Bulletin 42: 1236–1263. 10.1016/S0025-326X(01)00240-5 11827109

[B21] Nei M Tajima F (1983) Maximum likelihood estimation of the number of nucleotide substitutions from restriction sites data. Genetics 105: 207–217. 6311668 10.1093/genetics/105.1.207 PMC1202146

[B22] Pons O Petit RJ (1996) Measuring and testing genetic differentiation with ordered vs. unordered alleles. Genetics 144: 1237–1245. 10.1093/genetics/144.3.1237 PMC1207615 8913764

[B23] Rambaut A (2014) FigTree 1.3. Available at: http:tree.bio.ed.ac.uk/software/figtree

[B24] Rambaut A Drummond AJ Suchard M (2013) Tracer v1.6. http://beast.bio.ed.ac.uk/Trace

[B25] Rozas J Sanchez-DelBarrio JC Messeguer X Rozas R (2003) DnaSP, DNA polymorphism analyses by the coalescent and other methods. Bioinformatics 19: 2496–2497. 10.1093/bioinformatics/btg359 14668244

[B26] Shi YF Cui ZJ Su Z (2006) The Quaternary glaciations and environmental variations in China. Hebei Science and Technology Press, Shijiazhuang.

[B27] Tamura K Stecher G Peterson D Filipski A Kumar S (2013) MEGA6: Molecular evolutionary genetics analysis version 6.0. Molecular Biology and Evolution 30: 2725–2729. 10.1093/molbev/mst197 24132122 PMC3840312

[B28] Tang KL Agnew MK Chen WJ Hirt MV Sado T Schneider LM Freyhof J Sulaiman Z Swartz E Vidthayanon C Miya M Saitoh K Simons AM Wood RM Mayden RL (2010) Systematics of the subfamily Danioninae (Teleostei: Cypriniformes: Cyprinidae). Molecular phylogenetics and Evolution 57: 189–214. 10.1016/j.ympev.2010.05.021 20553898

[B29] Templeton AR (1993) The ‘Eve’ hypothesis: a genetic critique and reanalysis. American Anthropologist 95: 51–72. 10.1525/aa.1993.95.1.02a00030

[B30] Thompson JD Gibson TJ Plewniak F Jeanmougin F Higgins DG (1997) The Clustal X windows interface: flexible strategies for multiple sequence alignment aided by quality analysis tools. Nucleic acids research 24: 4876–4882. 10.1093/nar/25.24.4876 PMC147148 9396791

[B31] Tzeng CS (1986) Distribution of freshwater fishes of Taiwan. Journal of Taiwan Museum 39: 127–146.

[B32] Voris HK (2000) Maps of Pleistocene sea levels in Southeast Asia: shorelines, river systems and time durations. Journal of Biogeography 27: 1153–1167. 10.1046/j.1365-2699.2000.00489.x

[B33] Wang CF Hsieh CH Lee SC Wang HY (2011) Systematics and Phylogeography of the Taiwanese endemic minnow *Candidia barbatus* (Pisces: Cyprinidae) based on DNA sequences, allozymic, and morphological analyses. Zoological Journal of the Linnean Society 161: 613–632. 10.1111/j.1096-3642.2010.00646.x

[B34] Xiao W Zhang Y Liu H (2001) Molecular systematics of Xenocyprinae (Teleostei: Cyprinidae): taxonomy, biogeography, and coevolution of a special group restricted in East Asia. Molecular Phylogenetics and Evolution 18: 163–173. 10.1006/mpev.2000.0879 11161753

[B35] Yang JQ Hsu KC Liu Z Su LW Kuo PK Tang WQ Zhou ZC Liu D Bao BL Lin HD (2016) The population history of *Garra orientalis* (Teleostei: Cyprinidae) using mitochondrial DNA and microsatellite data with approximate Bayesian computation. BMC Evolutionary Biology 16: 73. 10.1186/s12862-016-0645-9 PMC4827224 27068356

[B36] Yang JQ Tang WQ Liao TY Sun Y Zhou ZC Han CC Liu D Lin HD (2012) Phylogeographical analysis on *Squalidus argentatus* recapitulates historical landscapes and drainage evolution on the island of Taiwan and mainland China. International Journal of Molecular Sciences 13: 1405–1425. 10.3390/ijms13021405 22408398 PMC3291967

[B37] Yap SY (2002) On the distributional patterns of Southeast-East Asian freshwater fish and their history. Journal of Biogeography 29: 1187–1199. 10.1046/j.1365-2699.2002.00771.x

[B38] Yu Y Harris AJ He X (2010) S-DIVA (statistical dispersal-vicariance analysis): a tool for inferring biogeographic histories. Molecular phylogenetics and Evolution 56: 848–850. 10.1016/j.ympev.2010.04.011 20399277

[B39] Zeng ZX Zeng XZ (1989) Physical geography of Hainan. Science Press, Beijing.

[B40] Zhang HN Chen CG Huang KR Li ZQ Zhang FL Chen GZ (1990) The new geological structures, tectonic movements and geological environment in coastal line of South China. Earthquake Press, Beijing (in Chinese).

[B41] Zhao HT Wang LR Yuan JY (2007) Origin and time of Qiongzhou Strait. Marine Geology and Quaternary Geology 27: 33–40.

[B42] Zhou TQ Lin D Hsu KC Kuo PH Wang WK Tang WQ Liu D Yang JQ (2017) Spatial genetic structure of the cyprinid fish *Onychostoma lepturum* on Hainan Island. Mitochondrial DNA Part A 28: 901–908. 10.1080/24701394.2016.1209193 27606601

[B43] Zhu Y Zhao Y Huang K (2013) *Aphyocypris pulchrilineata*, a new miniature cyprinid species (Teleostei: Cypriniformes: Cyprinidae) form Guangxi, China. Ichthyological research 60: 232–236. 10.1007/s10228-013-0338-y

